# Treatment of Prosthetic Vascular Graft Infections Using Cryopreserved Homografts: A Surgical Case Series

**DOI:** 10.7759/cureus.94183

**Published:** 2025-10-09

**Authors:** Roman Kamorov, Maxim I Tkachev, Abubakar I. Sidik, Maxim L Khavandeev, Ivan Karpenko, Malik K Al-Ariki, Md Limon Hossain, Vladislav V Dontsov

**Affiliations:** 1 Cardiovascular Surgery, I.M. Sechenov First Moscow State Medical University, Moscow, RUS; 2 Cardiovascular Surgery, Peoples' Friendship University of Russia, Moscow, RUS; 3 Cardiovascular Surgery, Gusak Institute of Emergency and Reconstructive Surgery, Donetsk, RUS; 4 Cardiothoracic Surgery, A.A. Vishnevskiy Hospital, Moscow, RUS; 5 Cardiothoracic Surgery, Peoples' Friendship University of Russia, Moscow, RUS; 6 Cardiology, I.M. Sechenov First Moscow State Medical University, Moscow, RUS; 7 Cardiothoracic Surgery, Moscow Regional Research and Clinical Institute, Moscow, RUS

**Keywords:** aorto-bifemoral bypass, homologous materials, postoperative complications, prosthetic infection, vascular homografts

## Abstract

Infections of vascular prostheses remain a formidable challenge in vascular surgery due to their high morbidity and mortality rates. This retrospective study presents the clinical experience of using vascular homografts in four complex cases of infected synthetic vascular prostheses treated between 2015 and 2020. Patients presented with varied complications, including duodeno-paraprosthetic fistula, aneurysmal degeneration, thromboembolism, and infection following aorto-bifemoral and iliofemoral bypasses, as well as stent-graft infections. In all but one case, homograft reconstruction led to favorable outcomes, with long-term follow-up confirming graft patency and absence of reinfection. One patient succumbed to sepsis and multiorgan failure postoperatively. The use of cryopreserved homografts demonstrated efficacy and safety, particularly in cases unsuitable for synthetic or autologous grafts. Our findings highlight the potential of vascular homografts as a valuable alternative in managing prosthetic graft infections, emphasizing the importance of individualized treatment approaches, multidisciplinary planning, and advanced surgical techniques.

## Introduction

Peripheral arterial disease (PAD) affects more than 200 million individuals globally and frequently necessitates surgical intervention to restore adequate blood flow and preserve limb function [1]. Synthetic vascular grafts are commonly utilized in revascularization procedures; however, they have a limited long-term patency, with only about 50% remaining functional at five years post-implantation [2]. Among the most serious complications following either elective or emergent vascular procedures is graft infection. Though infrequent, occurring in 0.2% to 5% of cases, these infections are associated with a high risk of mortality (25-88%) and limb loss (up to 60%) [3].

In aorto-femoral graft infection, treatment usually involves surgical intervention (with cryopreserved arterial allografts, autologous vein grafts, or antibiotic-impregnated synthetic grafts), often combined with antibiotic therapy [4]. Inspired by the use of homografts in cardiac surgery, particularly for treating infective endocarditis of the aortic valve, vascular surgeons have begun exploring homografts as a viable option for managing infected prosthetic grafts. These biological conduits offer the advantage of improved resistance to infection and are suitable for in-situ reconstruction. Although initial outcomes are encouraging, concerns remain about long-term durability and the influence of different preservation techniques. Also, challenges such as limited availability, variability in preservation protocols, and the absence of standardized clinical guidelines for the use of vascular homografts persist [3].

Optimal antibiotic therapy in aorto-femoral graft infections is limited by biofilm formation, poor tissue penetration, and resistant pathogens, making eradication difficult despite prolonged treatment. Cryopreserved arterial homografts are appealing as they resist biofilm formation, allow revascularization, and reduce reliance on long-term antibiotic suppression, offering superior infection control in contaminated fields. They also lower reinfection risk because they are biological tissues with preserved endothelial and extracellular matrix structures that integrate with host tissue and resist bacterial adhesion. Unlike synthetic grafts, they lack artificial surfaces for biofilm formation, and unlike autologous veins, they are larger-caliber, better matched, and less prone to degeneration in infected fields [4].

The primary goal of this study was to evaluate the safety and effectiveness of in-situ reconstruction using cryopreserved vascular homografts in patients with infected synthetic arterial grafts. Given the limited data on this complex clinical scenario, especially in the context of emerging alternative treatment options, our case series aims to provide valuable insights into clinical outcomes, complications, and mid-term prognosis associated with this surgical approach. 

## Case presentation

This retrospective, single-center case series included four patients with infected synthetic arterial grafts treated between 2015 and 2020 at Sechenov University. Diagnosis was based on imaging, clinical signs, and laboratory markers. All underwent graft removal and in-situ reconstruction using cryopreserved homografts, selected due to infection severity and lack of suitable autologous veins. Postoperative follow-up involved clinical, imaging, and laboratory monitoring. A multidisciplinary team managed all cases.

Case 1: Redo aorto-bifemoral bypass with a homograft due to a duodenal-paraprosthetic fistula

A 68-year-old male was admitted with worsening abdominal and lumbar pain, fever, general weakness, and rectal bleeding. Two days earlier, he had experienced a gastrointestinal hemorrhage. His medical history included occlusive atherosclerosis of the aorto-femoral segment, intermittent claudication, rest pain, and an ABFB with a synthetic graft performed five years prior.

Duplex ultrasound showed a para-aortic hematoma and deformation of the existing graft. Contrast-enhanced CT confirmed a para-aortic hematoma extending into the retroperitoneal space and identified a duodenal-paraprosthetic fistula. Laboratory tests revealed anemia with hemoglobin of 95 g/L (reference: 130-170 g/L), leukocytosis of 14.2 × 10⁹/L (reference: 4.0-9.0 × 10⁹/L), elevated erythrocyte sedimentation rate of 45 mm/h (reference: <15 mm/h), and C-reactive protein of 68 mg/L (reference: <5 mg/L). Laboratory tests demonstrated leukocytosis, elevated ESR and C-reactive protein, and anemia, consistent with infection and inflammation.

**Figure 1:**
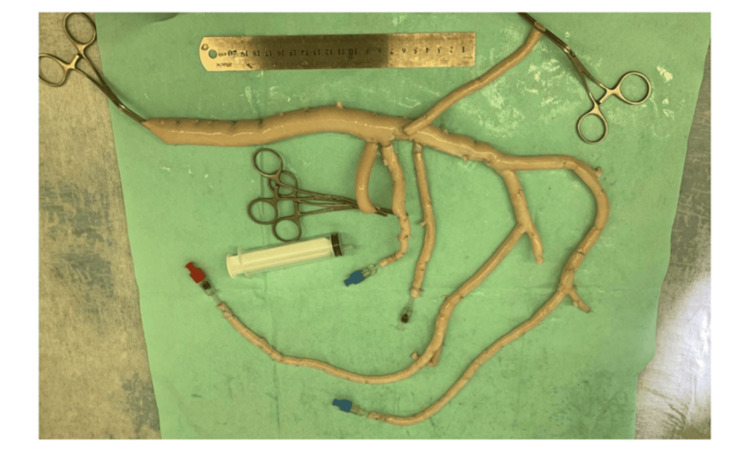
Intraoperative view of a redo aorto-bifemoral bypass with a cryopreserved homograft performed for a duodeno-paraprosthetic fistula.

The patient underwent a redo ABFB using a cryopreserved vascular homograft (Figure 1). Through midline laparotomy, dense duodenal infiltration adherent to the graft was identified. Careful dissection revealed a 4-mm fistula between the duodenum and graft. The duodenal defect was repaired with double-layer interrupted Vicryl 4/0 sutures without requiring intestinal resection. Bilateral femoral access facilitated excision of old scars and mobilization of vessels. After administering 5000 IU of heparin, the infected graft was excised. A proximal anastomosis between the aorta and homograft was created using Prolene 4/0. Homograft limbs were tunneled beneath the inguinal ligament and anastomosed end-to-side to the common femoral arteries with Prolene 5/0 sutures. Blood flow was successfully restored, confirmed by the strong pulsation of the homograft limbs.

On postoperative day seven, rebleeding due to suture dehiscence from the proximal anastomosis necessitated revision surgery. Partial suture dehiscence was repaired and reinforced with a synthetic patch. Cultures and postoperative imaging revealed no reinfection. At eight-year follow-up, CT and duplex ultrasound demonstrated patent grafts without occlusion or infection, confirming excellent long-term stability.

Case 2: Aorto-femoral bypass with a homograft due to prosthetic graft thrombosis

A 66-year-old male with atherosclerosis of the aorto-femoral and femoropopliteal segments underwent ABFB with a PTFE graft in 2015. Two years later, he required reoperation for thrombectomy of the left graft limb due to acute thrombosis, causing limb ischemia. One month post-thrombectomy, he developed worsening rest pain in the left leg, numbness, and coldness.

Ultrasound and CT angiography showed occlusion of the left graft limb, absent flow in the popliteal artery, and a distal anastomotic aneurysm (3.5 × 3.0 cm) with intraluminal thrombosis. Physical examination revealed erythema and tenderness in the left groin. Laboratory tests revealed leukocytosis of 12.8 ×10⁹/L (reference: 4.0-9.0 ×10⁹/L), elevated ESR of 38 mm/h (reference: <15 mm/h), and C-reactive protein of 52 mg/L (reference: <5 mg/L). Laboratory results indicated leukocytosis and elevated ESR and CRP, suggestive of infection.

**Figure 2:**
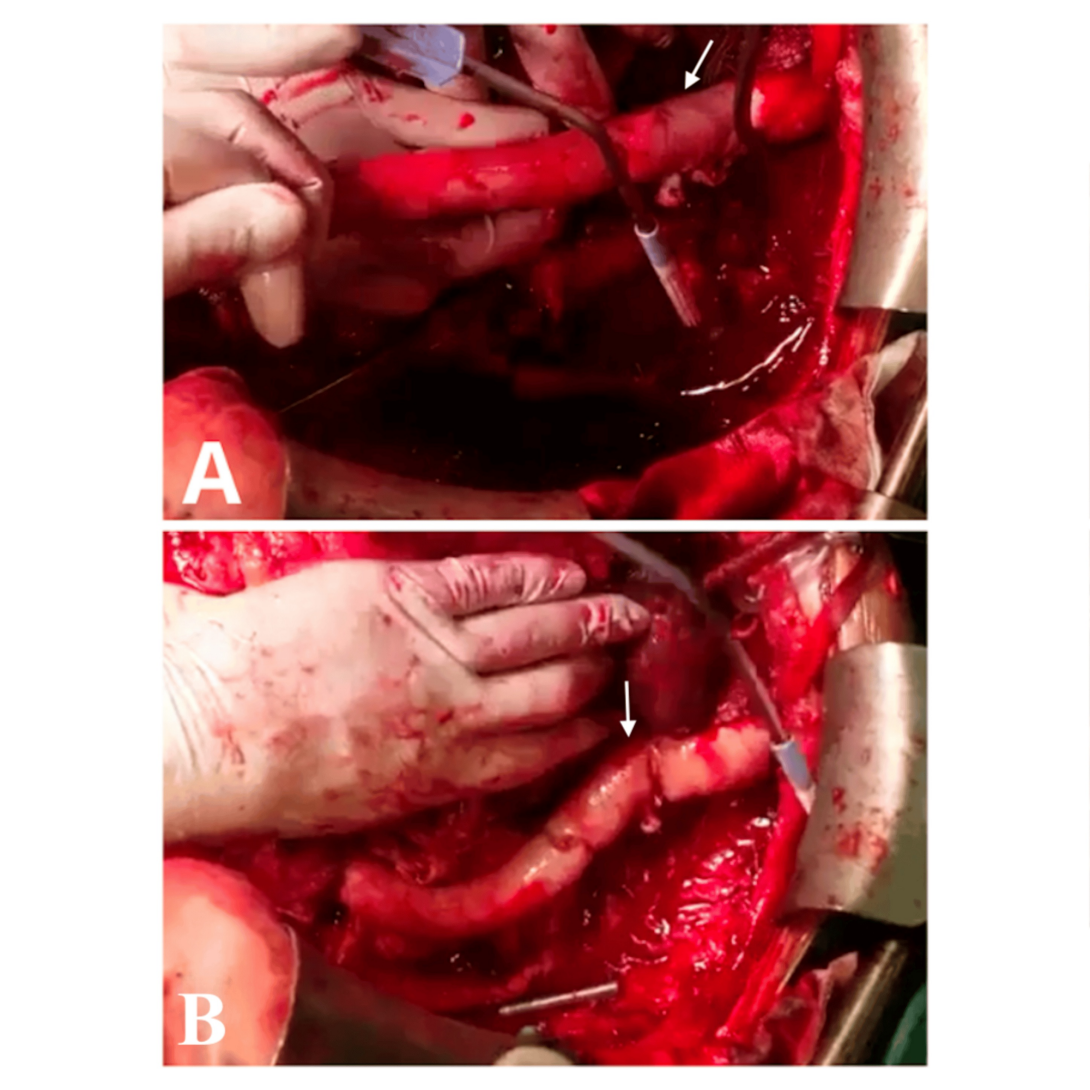
Aorto-femoral bypass with a homograft due to prosthetic graft thrombosis A. Occluded synthetic graft with distal anastomotic aneurysm (white arrow). B. Intraoperative replacement with a cryopreserved homograft for aorto-femoral bypass (the white arrow shows the new anastomotic site).

A linear aorto-deep femoral bypass was performed using a cryopreserved homograft. Midline laparotomy exposed the aorta and iliac arteries, while a left groin incision isolated the femoral artery. The infected perigraft tissue was excised, and the left limb of the synthetic graft was removed. The homograft was anastomosed proximally to the aortic stump and distally to the deep femoral artery (Figures 2A, 2B). Seven years later, follow-up ultrasound and CT confirmed the homograft’s patency with no evidence of infection or occlusion.

Case 3: Redo aorto-bifemoral bypass with a homograft due stent-graft because of stent-graft infection

In 2020, a 63-year-old patient presented with worsening abdominal pain, fever, chills, and weakness, occurring two years after stenting of the abdominal aorta and both common iliac arteries (2018). CT imaging revealed a 6×8 cm periprosthetic abscess extending into the retroperitoneal space, with destruction of the aortic wall at the stent-graft site and significantly reduced distal aortic and iliac artery patency. Preoperative blood cultures grew *Staphylococcus aureus*, sensitive to vancomycin and ceftriaxone. Laboratory tests demonstrated leukocytosis of 15.6 × 10⁹/L (reference: 4.0-9.0 × 10⁹/L), anemia with hemoglobin of 102 g/L (reference: 130-170 g/L), elevated ESR of 55 mm/h (reference: <15 mm/h), and C-reactive protein of 96 mg/L (reference: <5 mg/L).

**Figure 3:**
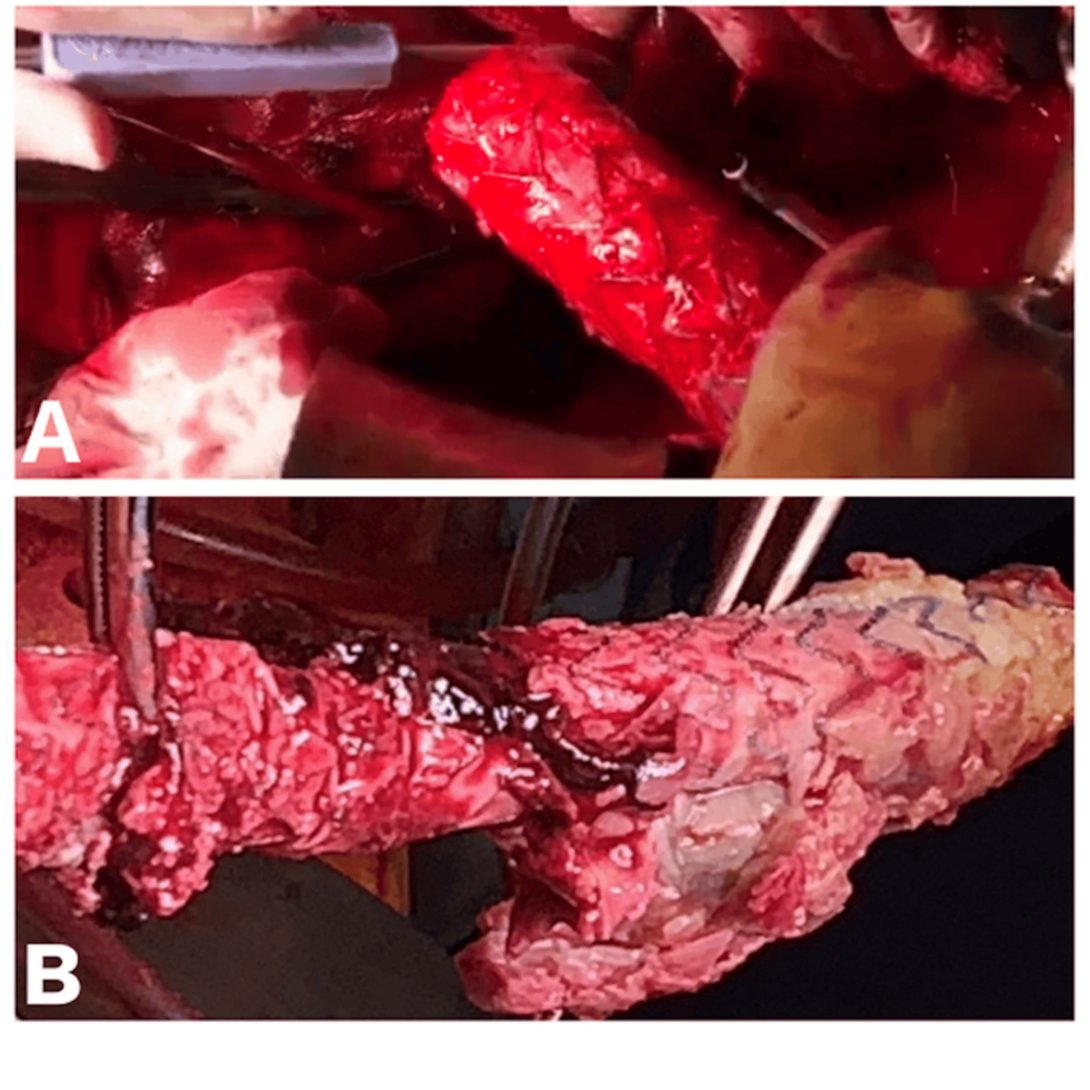
Redo aorto-bifemoral bypass with a homograft due stent-graft due to stent-graft infection A, B. Explanted infected bifurcated stent-graft encased in abscess tissue.

Surgical access exposed an infected bifurcated stent-graft encased in an abscess. The cavity was irrigated with chlorhexidine solution, and purulent material, necrotic tissue, and the infected stent-graft were removed (Figures 3A, 3B). Reconstruction was performed with an ABFB using a vascular homograft.

Postoperatively, the patient experienced recurrent fevers requiring intravenous vancomycin and ceftriaxone for four weeks. After discharge, oral antibiotics continued for three months with close outpatient monitoring. At four-year follow-up, contrast-enhanced CT showed the homograft remained patent with no evidence of infection or occlusion.

Case 4: Redo aorto-bifemoral bypass with homograft

A 65-year-old patient was admitted in 2018 with persistent rest pain in the left foot, worsening at night, and calf pain after walking 50-100 meters. Examination showed acute arterial insufficiency of the left lower limb: pale, cold skin with reduced local temperature amid systemic hyperthermia. Pulsation in the left femoral artery was weak, and distal pulses (dorsalis pedis, posterior tibial) were absent; right-sided pulses were intact. His history included hypertension and type 2 diabetes mellitus.

Five months earlier, the patient underwent ABFB with a Dacron graft for aorto-iliac occlusion. Ultrasound confirmed occlusion of the graft’s left branch, with surrounding inflammatory changes. CT angiography revealed periprosthetic infection and elevated inflammatory markers (leukocytosis, CRP). Laboratory tests showed leukocytosis of 16.4 × 10⁹/L (reference: 4.0-9.0 × 10⁹/L), anemia with hemoglobin of 108 g/L (reference: 130-170 g/L), elevated ESR of 60 mm/h (reference: <15 mm/h), and C-reactive protein of 120 mg/L (reference: <5 mg/L).

**Figure 4:**
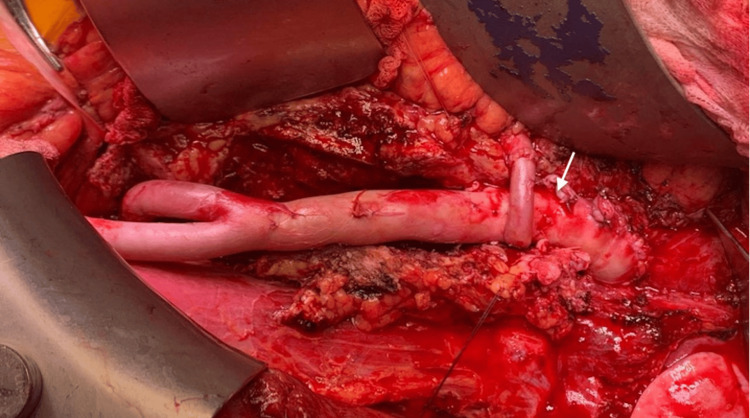
Intraoperative view of redo aorto-bifemoral bypass with homograft after removal of an infected Dacron graft (the white arrow shows the anastomotic site).

A relaparotomy was performed. The previous graft was entirely removed, and infected tissue and pus were debrided. Pus cultures grew *Pseudomonas aeruginosa*. A redo ABFB was performed using a homograft, anastomosed proximally to the aorta below the renal arteries and distally to the common femoral arteries bilaterally (Figure 4). Postoperatively, the patient developed multiple organ failure and sepsis despite intensive care. He died on day six in the ICU.

## Discussion

Vascular homografts represent a promising alternative to synthetic prostheses in vascular surgery, particularly in complex clinical situations where synthetic materials may be insufficiently effective or carry an increased risk of complications. The relative availability of homografts and their biological compatibility open up new opportunities for vascular reconstruction, allowing surgeons to apply them in cases that were previously considered untreatable or requiring more complex approaches. Infections of vascular prostheses, despite their low frequency, remain a serious problem and challenge for all vascular surgeons. Even though several methods of treating this complication have been proposed, such as in situ reconstruction using synthetic grafts or extra-anatomical prosthetic grafting, mortality and amputation rates remain high [3,5].

Cryopreserved arterial allografts demonstrate the lowest reinfection rates, around 5-10%, with 2-5-year patency of 70-80%, offering excellent infection resistance, though their availability is limited. Autologous vein grafts (neo-aortoiliac systems) have slightly higher reinfection rates of 8-15% and patency between 70-85%, but the procedure is technically demanding and may cause leg swelling. Antibiotic-bonded prostheses achieve good patency (>80%) but carry a higher reinfection risk of 20-30%. Silver-coated grafts show reinfection rates of 20-25% with 70-80% patency, serving as a useful alternative when homografts are not available [4]. Homografts are less prone to infection than prosthetic grafts, which allows for in situ replacement with a low risk of reinfection and reduces the risk of aortic stump rupture or occlusion of extra-anatomical prosthetic shunts. However, occlusion and late degeneration of the homograft can be expected due to preservation methods and chronic rejection [3,5].

Vogt et al. analyzed the surgical outcomes of 72 patients with mycotic aneurysms and/or infected vascular prostheses. A synthetic conduit was used in 38 cases and cryopreserved arterial homografts in 34 patients. The choice of material depended on the availability of the homograft at the time of surgery. Cryopreserved homografts demonstrated significantly higher survival, shorter hospital stays, shorter durations of postoperative antibiotic therapy, and lower complications than traditional surgical methods; additionally, the economic costs in the homograft group were significantly lower [5]. In our study, the effectiveness of cryopreserved homografts applied in our practice for infectious complications after previous surgical interventions was confirmed.

In the study by Kieffer et al., the early and long-term outcomes of replacing an infected graft with a homograft were comparable to other methods of treating infrarenal aortic graft infections [6]. Our results confirm the effectiveness of using cryopreserved arterial homografts in the treatment of infectious complications of vascular prostheses.

Infection of vascular prostheses implanted in the abdominal cavity is a serious problem, despite its relatively low incidence (0.3-5% of all implantations). The three treatment options are conservative treatment with long-term antibiotic therapy, removal of the graft and performance of extra-anatomical axillo-(bi)femoral bypass, and in situ graft replacement, using either great saphenous veins (GSV), synthetic prostheses, or homografts. Arterial reconstruction with a new synthetic prosthesis is associated with a high risk of reinfection, which is mitigated by soaking the prosthesis in rifampicin and/or using silver-coated prostheses. Alternatively, an intraoperative graft can be made from the GSV, which is more resistant to infection. However, the use of GSV is associated with the formation of aneurysms and stenoses [4]. We do use GSV and try as much as possible to avoid extra-anatomical axillo-femoral bypass, considering the procedure non-physiological.

A meta-analysis of 1377 patients who underwent homograft implantation for prosthetic infection revealed a 30-day mortality rate of 15%; cumulative graft-related mortality was 3.6%; degeneration occurred in 5% of cases; rupture in 6%; 25% required reoperations, and 3.8% of patients had amputations [7].

In a retrospective analysis of 200 patients who received cryopreserved homografts after removal of an infected graft, the 30-day mortality was 11%. Reoperation due to homograft issues was required in 59 patients, with 15 resulting in death. After six months, 25 patients experienced long-term complications such as rupture, pseudoaneurysm, aneurysm, thrombosis, and stenosis, requiring reoperation and resulting in one fatal outcome. The five-year survival rate was 56%, with a reoperation rate of 30%, limb preservation rate of 89%, primary patency of 80%, and a 12% re-infection rate. The authors report that long-term complications of homografts are common, but they are associated with low mortality and amputation rates [8]. In contrast, we did not observe severe complications like homograft rupture or aneurysmal dilation during the follow-up period. Our hospital results included one fatality on the sixth day. In another case, on the seventh day of hospitalization, rebleeding occurred at the proximal anastomosis, which was successfully managed surgically.

A comparative analysis of methods for treating aortic graft infections, including extra-anatomical bypass, rifampicin-coated prostheses, cryopreserved allografts, and autogenous veins in a meta-analysis of 37 studies, showed differences in complication rates. The average incidence of adverse events was 16% for extra-anatomical bypass, 7% for rifampicin-coated prostheses, 9% for cryopreserved allografts, and 10% for autogenous veins. The lowest complication rate was observed with rifampicin-coated prostheses, indicating their high effectiveness. These data challenge the use of extra-anatomical bypass as the gold standard for treating aortic graft infections and highlight the need for further research to optimize clinical practice [4]. In our practice, we do not use extra-anatomical bypass for treating aortic graft infections. Additionally, we remain cautious about the repeated use of prosthetic grafts due to concerns about infection recurrence and complications.

This case series has limitations. First, the small sample size of four patients limits the generalizability of our findings and precludes statistical analysis. Second, the retrospective single-center design may introduce selection bias and restrict the ability to compare outcomes with alternative treatment strategies. Third, the follow-up duration varied among patients, and although most demonstrated favorable mid- to long-term results, larger studies with standardized follow-up are needed to assess long-term durability. Finally, the lack of a control group prevents direct comparison between cryopreserved homografts and synthetic grafts or autologous vein conduits in the management of prosthetic vascular graft infections.

## Conclusions

Vascular homografts are a promising alternative for treating infected stent-grafts, offering high biocompatibility, reduced reinfection and thrombosis risks, and favorable mid-term outcomes. Despite technical challenges and limited availability, their use shows potential in selected cases. Further research is needed to optimize preservation methods, assess long-term durability, refine patient selection, and integrate advanced imaging for early complication detection.
